# The Colleges

**Published:** 1882-10

**Authors:** 


### The Colleges.

The time for opening the Dental Colleges is at hand, and the voice of the student is heard in the land.

A great responsibility rests upon our professional schools; this is true of all, but especially of the dental schools. As a profession dentistry is in its infancy, the developing stage; and certainly those whose province it is, to in any way, give direction to this development, have a charge that cannot be lightly esteemed. The great effort of teachers should be to secure the highest and best advancement of those committed to their charge, and *well* fit them for the great responsibilities of their future work, rather than to try to bring into the respective schools the largest possible number of students.

Great care should be exercised in the colleges, as well as with the members of the profession, as to giving encouragement to those who are unprepared, or who are obviously wanting in natural endowments. Many such have sought to enter the profession; some by one way, and some by another. Most certainly the Colleges should see to it that persons without preparation or adaptation, are not encouraged to enter as students; in too many cases such have only learned the truth after a long and laborious effort, and loss of time and money, which might have been saved by a little timely advice.

Several dental colleges have been organized within the last year or two; and this is well if they are established because of a demand for more and better instruction, which we hope is the case. The older schools have some advantage, from the fact of being known and having established reputation; but the new schools have the advantage of starting upon a higher plain than those first established. They may, if they will, keep out of ruts and mere routine methods, and avoid errors and impracticable things that have often embarrassed our schools. They can profit by the experience of those that have gone before. May they all do it; and may every dental college in the country do better work, and enjoy a higher prosperity than ever before.

